# Preparing Vegetable Tissues for the Microscope

**Published:** 1875-01

**Authors:** 


*Preparing Vegetable Tissues for the Microscope—A New Discovery.*—Dr. W. G. Harrison, of the Maryland Academy of Sciences, reports that the committee of biology and microscopy, while pursuing the subject of improvement in preparing and mounting subjects for the microscope, discovered a new mode of preparing fresh vegetable tissues—a discovery of much importance. This process may be briefly described as follows: First, the specimen, say a leaf, is decolorized by soaking in a solution of chlorinate of soda. Second, the soda remaining in the leaf is removed by washing in water. Third, the leaf is then washed, first in dilute alcohol, increasing its strength gradually until the water is removed. Fourth, the specimen is then submitted to one of the staining fluids known to the art; analine blue is generally used, and was selected for the slide shown by the doctor. If too much color is given, it can be partially removed by alcohol. Fifth, a solution of oil of cloves is then used to remove the alcohol. Sixth, the leaf being properly prepared, is mounted with Canada balsam.

